# Tuning Protein Hydrogel Mechanics through Modulation of Nanoscale Unfolding and Entanglement in Postgelation Relaxation

**DOI:** 10.1021/acsnano.2c02369

**Published:** 2022-06-22

**Authors:** Matt D. G. Hughes, Sophie Cussons, Najet Mahmoudi, David J. Brockwell, Lorna Dougan

**Affiliations:** 1 School of Physics and Astronomy, Faculty of Engineering and Physical Sciences, University of Leeds, Leeds LS2 9JT, U.K.; 2 Astbury Centre for Structural Molecular Biology, University of Leeds, Leeds LS2 9JT, U.K.; 3 School of Molecular and Cellular Biology, Faculty of Biological Sciences, University of Leeds, Leeds LS2 9JT, U.K.; 4 ISIS Neutron and Muon Spallation Source, STFC Rutherford Appleton Laboratory, Oxfordshire OX11 0QX, U.K.

**Keywords:** protein-hydrogel, biomimetic, bioinspired, chemical responsive hydrogel, rheology, entanglement, protein unfolding

## Abstract

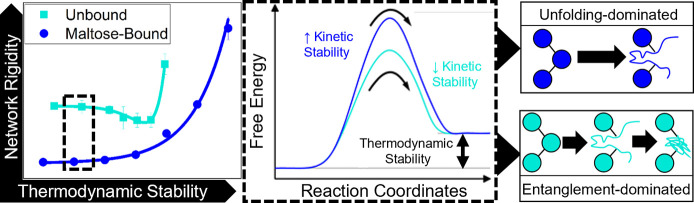

Globular folded proteins are versatile nanoscale building blocks to create biomaterials with mechanical robustness and inherent biological functionality due to their specific and well-defined folded structures. Modulating the nanoscale unfolding of protein building blocks during network formation (*in situ* protein unfolding) provides potent opportunities to control the protein network structure and mechanics. Here, we control protein unfolding during the formation of hydrogels constructed from chemically cross-linked maltose binding protein using ligand binding and the addition of cosolutes to modulate protein kinetic and thermodynamic stability. Bulk shear rheology characterizes the storage moduli of the bound and unbound protein hydrogels and reveals a correlation between network rigidity, characterized as an increase in the storage modulus, and protein thermodynamic stability. Furthermore, analysis of the network relaxation behavior identifies a crossover from an unfolding dominated regime to an entanglement dominated regime. Control of *in situ* protein unfolding and entanglement provides an important route to finely tune the architecture, mechanics, and dynamic relaxation of protein hydrogels. Such predictive control will be advantageous for future smart biomaterials for applications which require responsive and dynamic modulation of mechanical properties and biological function.

Biomacromolecules provide a wealth of evolutionary-optimized building blocks for the development and design of biomimetic and bioinspired materials. Such materials include hydrogels: cross-linked networks of hydrophilic building blocks, swollen by large volumes of water.^1−4^ Folded proteins have emerged as attractive hydrogel components due to their intrinsic stability, specific well-defined structure, and inherent biological functionality.^5^ The folded structures of single proteins have been found to be mechanically robust with well-defined mechanical characteristics.^6−10^ Over the last several decades, many detailed single-molecule studies have produced a wealth of mechanically well-characterized proteins.^11−15^ This information has been used to develop folded protein-based hydrogels^16,17^ with interesting mechanical properties such as mimicking tissues and scaffolds,^18,19^ regulating size and shape,^20,21^ and defining bulk mechanical properties, e.g., brittleness.^22^ In addition to their mechanical characteristics, the inherent biological functionality of proteins has allowed for the design of smart biomaterials in which the network exhibits inherent biofunctionality becoming mechanically responsive to stimuli including, but not limited to ionic strength,^23^ redox environment,^24−26^ and illumination by light.^27^

Changing protein stability can be used to tune hydrogel properties. We previously demonstrated that the use of ligand-bound maltose bound protein (MBP), which has increased thermodynamic and kinetic stability, resulted in a protein network with increased mechanical rigidity, measured as an increase in the storage modulus, while retaining the network architecture.^28^ Interestingly, the translation of mechanics across scales was surprisingly nonlinear with only ∼30% of the more stable bound-MBP required to achieve a 1.67-fold increase in network rigidity. Li et al. designed a *de novo* ferredoxin-like protein, with a low unfolding force (∼5 pN), to undergo force-induced unfolding of the protein domain during photochemical gelation.^29^ This resulted in a highly elastic and tough hydrogel that could withstand strains of 450% before breaking. More recently, we exploited nanostaples (intraprotein disulphide bonds) to restrict protein unfolding, allowing the control of network architecture and mechanics.^30^ This work was important because it highlighted the potential of manipulating protein unfolding to predictively control protein hydrogel properties, for example, the ability to program shape memory into biomaterials; Popa et al. exploited BSA hydrogels and the addition of the denaturant guanidium to reversibly unfold and refold the protein, resulting in gels that “remembered” bulk 3D shapes after large deformations to the material.^31^ The authors also demonstrated that this bulk shape memory could be programmed into BSA-based hydrogels by modulating the stability of BSA through the presence of Ni^2+^ and Cu^2+^ ions.^20^ More recently, Li et al. used a hydrophobic peptide–hydrophilic protein copolymer to construct a thermo-responsive hydrogel.^32^ Heating (cooling) this copolymer hydrogel results in reversible aggregation (or relaxation) of the hydrophobic peptides, causing an increase (or decrease) in the Young’s Modulus of the protein-copolymer hydrogel, demonstrating how the reversible collapse and uncoiling of hydrogel building blocks can control mechanical properties.

Despite these successes, the physics underlying the formation and relaxation of protein networks is still poorly understood. During gelation of protein hydrogels, protein building blocks are exposed to forces on the order 10^29^–100 pN.^28^ Previous studies of stress relaxation in soft matter and biological systems have provided rich information on the underlying physics. Such an approach could therefore shed light on protein hydrogel network relaxation and the role of internal stresses caused by gelation. Recently Gong et al., using a tough self-healing polyampholyte hydrogel, showed that the gel exhibited two distinct relaxation times: one on the order of 10 s of seconds due to the relaxation of chain segments (∼1 nm) and the other on the order of 100 s due to the phase structure of the network (∼100 nm).^33^ Similarly, stress relaxation measurements of alginate gels have shown distinctive relaxation times due to the ionic and covalent cross-linking motifs exhibit strikingly different stress relaxation behavior.^34,35^ Motivated by the understanding of stress relaxation developed in other soft matter systems,^34−36^ combined with previous characterization of protein hydrogel relaxation,^28,30^ we hope to gain insight into the role of protein unfolding in the relaxation of folded protein hydrogels. Furthermore, we seek to understand how protein thermodynamic and kinetic stabilities alter the relaxation behavior and, ultimately, the mechanical properties of the mature hydrogel.

Here, we have utilized hydrogels constructed from the protein MBP^28^ as a model system to explore the importance of protein stability on network formation and relaxation. To achieve this, we manipulate the thermodynamic stability of both unbound-MBP (U-MBP) and the kinetically more stable maltose-bound MBP (MB-MBP) through the addition of increasing concentrations of the denaturant urea. MBP has been extensively characterized; thermodynamically,^37^ chemically,^38^ and mechanically.^39,40^ A combined experimental approach of differential scanning calorimetry (DSC) and shear rheology demonstrates that thermodynamic and kinetic stabilities of the protein have distinct effects on the postgelation relaxation behavior, leading to dramatic changes in the rigidity and formation process of protein hydrogel networks.

## Results and Discussion

### Building Block Selection and Modulation of Stabilities on the Molecular Level

**Figure 1:**
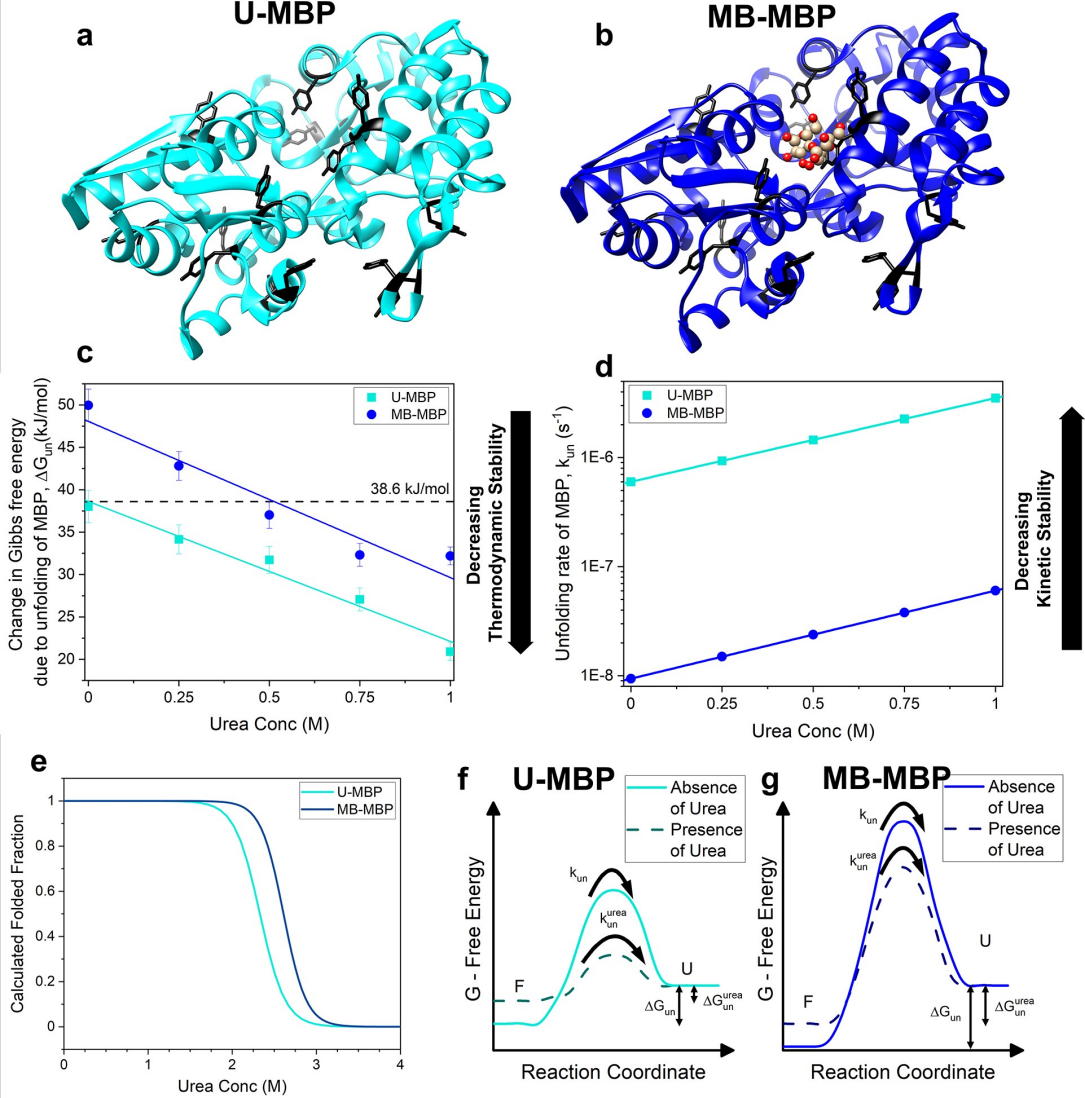
Crystal structures of MBP in the (a) absence (light blue, PDB code: 1JW5) and (b) presence (dark blue, PDB code: 1Y4C) of maltose (red ball and stick representation). The cross-linking tyrosine residues are colored black. (c) Gibbs free energy of unfolding of MBP (concentration: 100 mg/mL) in the absence (light blue) and presence (dark blue) of maltose as determined by DSC, at varying concentrations of urea,^44^ where the number of repeats per urea concentration, *N* = 3. Solid lines represent linear fits consistent with established linear relationship between Δ*G*_un_ and urea concentration. Black horizontal line represents the fitted value of thermodynamic stability of MBP in the unbound state in the absence of denaturant. (d) Calculated unfolding rates of MBP as a function of urea concentration in the absence and presence of maltose. The values for *m*_u_ and *k*_un_([urea] = 0M) taken from ref (38) were 0.96 ± 0.04 kcal·mol^–1^ and (6 ± 1) × 10^–7^ s^–1^, respectively in the unbound state and 1.01 ± 0.03kcal·mol^–1^ and (9 ± 3) × 10^–9^ s^–1^, respectively in the bound state. (e) Calculated folded fraction of U-MBP (light blue) and MB-MBP (dark blue) as a function of urea concentrations. Curves were calculated from values extracted from DSC data in Figure 1c. (f,g) Schematics of a two-state unfolding energy landscape of U-MBP and MB-MBP respectively, where the protein transition from a folded state (F) over an energy barrier to an unfolded state (U). Δ*G*_un_ and Δ*G*_un_^urea^ are the free energy differences between the folded and unfolded states in the absence and presence of urea respectively, while *k*_un_ and *k*_un_^urea^ are the rates at which the protein transitions over the energy barrier in the absence and presence of urea, respectively.

MBP was selected as a model system to investigate the differential role of building block thermodynamic and kinetic stabilities in defining the architectural and mechanical properties of a hierarchical protein hydrogel network. The 370-residue protein is a transport protein in *Escherichia coli* for the ligand maltose, containing 14 solvent exposed tyrosine residues (allowing formation of gel network^41^ via residue specific photochemical cross-linking) and is highly expressing (∼300 mg per liter of cell culture). X-ray crystallography has been performed on MBP in the absence^42^ and presence^43^ of its ligand maltose to determine the crystal structure of the protein (Figure 1a,b). The structure of MBP comprises two lobes connected by a hinge region (Figure 1a,b), which shows no significant difference in size or shape (RMSD = 4 Å) upon the binding of maltose.

MBP is an ideal model system for two key reasons: First, MBP has been previously characterized as an effective hydrogel building block.^28^ Second, the binding of a single maltose molecule to MBP causes an increase in the thermodynamic stability of the protein,^37^ measured as a 21% increase in the change in the Gibbs free energy going from the folded state to the unfolded state, Δ*G*_UN_ (Figure 1c). Furthermore, maltose-bound MBP exhibits a higher energy barrier to unfolding, characterized by a decrease in the unfolding rate of MBP^38^ (Figure 1d), hence an enhanced kinetic stability.

To investigate the effects of protein thermodynamic and kinetic stabilities on the overall properties of a cross-linked network of folded protein, the protein stability can be varied and controlled. In this work, we achieve this through the addition of the denaturant urea. Urea is known to destabilize folded proteins by lowering their thermodynamic stability^45,46^ and increasing their unfolding rate^38,47,48^ (decreasing kinetic stability) and in high concentration causes proteins to unfold completely.

DSC was employed to determine the change in Δ*G*_UN_ of U- and MB-MBP in increasing concentrations of urea. The thermodynamic stability was determined by measuring the “melting” temperature of the MBP fold (i.e., the median temperature at which the protein transitions from a folded state to an unfolded state) and the associated enthalpy change from which the Δ*G*_UN_ of the protein could be calculated. MBP has been observed to exhibit a single melting peak^28,37^ (Figure S1) suggesting a single unfolding event and a two-state unfolding. Figure 1c shows how Δ*G*_UN_ of U-MBP and MB-MBP varies as a function of urea concentration. As expected, the increasing concentration of urea decreases the Δ*G*_UN_ of both bound and unbound MBP, and this decrease is observed to be linear.^44^ Fitting this trend with a linear function allows us to extract a destabilizing coefficient of 16 ± 2 and 18 ± 3 kJ·mol^–1^ M ^–1^ for U- and MB-MBP, respectively. The destabilization coefficient of urea, known as the *m*-value, is understood to be related to the solvent-accessible surface area of a globular protein.^44^ Comparing the destabilizing coefficients of U- and MB-MBP, it can be seen they are within error of each other, implying that each has similar solvent-exposed surface area. This agrees with SAXS data (Figure S2), which shows no significant difference in the size and shape of MBP with or without bound maltose. Recent work on bilayered protein hydrogels constructed from elastomeric proteins has shown that small concentrations of denaturants actually have a stabilizing effect on the thermodynamic stability of the protein fold.^49^ From our DSC data we do not observe such a stabilizing effect on the thermodynamic stability of U- or MB-MBP over the range of urea concentrations considered. In addition to reducing the thermodynamic stability of proteins, urea also lowers the energy barrier to unfolding and hence reduces the kinetic stability of proteins, as demonstrated using pulse proteolysis by Na et al.^38^Figure 1d shows the unfolding rate of U-MBP and MB-MBP over the relevant range of urea concentrations, calculated using values for *m*_u_ and *k*_un_([urea] = 0 M) extracted by Na et al.^38^ The addition of urea increases the rate of unfolding of the protein and hence decreases the kinetic stability of both forms of the protein (Figure 1d). Determination of the kinetic and thermodynamic stability of U-MBP and MB-MBP allows us to calculate the fraction of folded MBP in differing amounts of urea. Figure 1e shows the calculated urea unfolding curve of U- and MB-MBP, showing that over our experimental range of urea concentrations there is no significant change in the fraction of folded protein in the solution pregelation.

It is important to note that while urea destabilizes both the thermodynamic and kinetic stability of MBP, the relative sizes of these destabilizing effects are very different in the presence or absence of maltose. For example, the binding of maltose causes a ∼21% increase in Δ*G*_UN_ of U-MBP, while the addition of 1 M urea leads a ∼42% reduction. This demonstrates that these effects are of similar magnitude (over the experimental relevant range of urea), meaning that urea can be used to alter both U- and MB-MBP to have the same thermodynamic stability (e.g., Δ*G*_UN_ of U-MBP in 0 M urea is equivalent to Δ*G*_UN_ of MB-MBP in ∼0.5 M urea). In contrast, the binding of maltose leads to a 60-fold decrease in the unfolding rate of MBP (increase in kinetic stability) which is an order of magnitude large than the 6-fold increase in unfolding rate (decrease in kinetic stability) in the presence of 1 M urea. This means that unlike the thermodynamic stability there is no urea concentration in our experimental range in which the unfolding rates of U-MBP and MB-MBP are equivalent, as MB-MBP will always exhibit a slower unfolding rate (enhanced kinetic stability) compared to U-MBP. Note that the addition of urea is also likely to reduce the mechanical unfolding force of both U-MBP and MB-MBP, as Li et al. has demonstrated that the addition of chemical denaturants linearly reduces the unfolding force of the model GB1 protein with denaturant concentration.^50^ Furthermore, the cross-linking of the MBP into a self-supporting network is also likely to affect the overall stability of the protein due to the internal gelation forces. We have previously estimated the internal gelation forces in U-MBP and MB-MBP hydrogels to be 70 ± 30 and 100 ± 40 pN, respectively,^28^ demonstrating that approximately identical internal forces are applied to both U-MBP and MB-MBP so we expect the contribution from cross-linking to be the same in both hydrogels.

By constructing hydrogels from either U-MBP or MB-MBP we can produce hydrogels with either enhanced or diminished protein kinetic stability (with marginal changes in thermodynamic stability) while simultaneously tuning the thermodynamic of the MBP building blocks by controlling the concentration of urea in the hydrogels. This molecular level dissection of protein “stability” is crucial in understanding the distinct roles played by protein thermodynamic and kinetic stability on the resultant higher order changes in the network and on the bulk properties of protein networks.

### Modulation of Network Rigidity on the Bulk Scale

To investigate the effects of protein thermodynamics and kinetic stabilities on the bulk mechanical properties of cross-linked networks of U-MBP and MB-MBP, we employed shear rheology. Pseudostrain-controlled rheology experiments were performed on photochemically cross-linked U-MBP and MB-MBP hydrogels (Materials and Methods) at a range of concentrations of urea, which is present in the sample during gelation.

**Figure 2:**
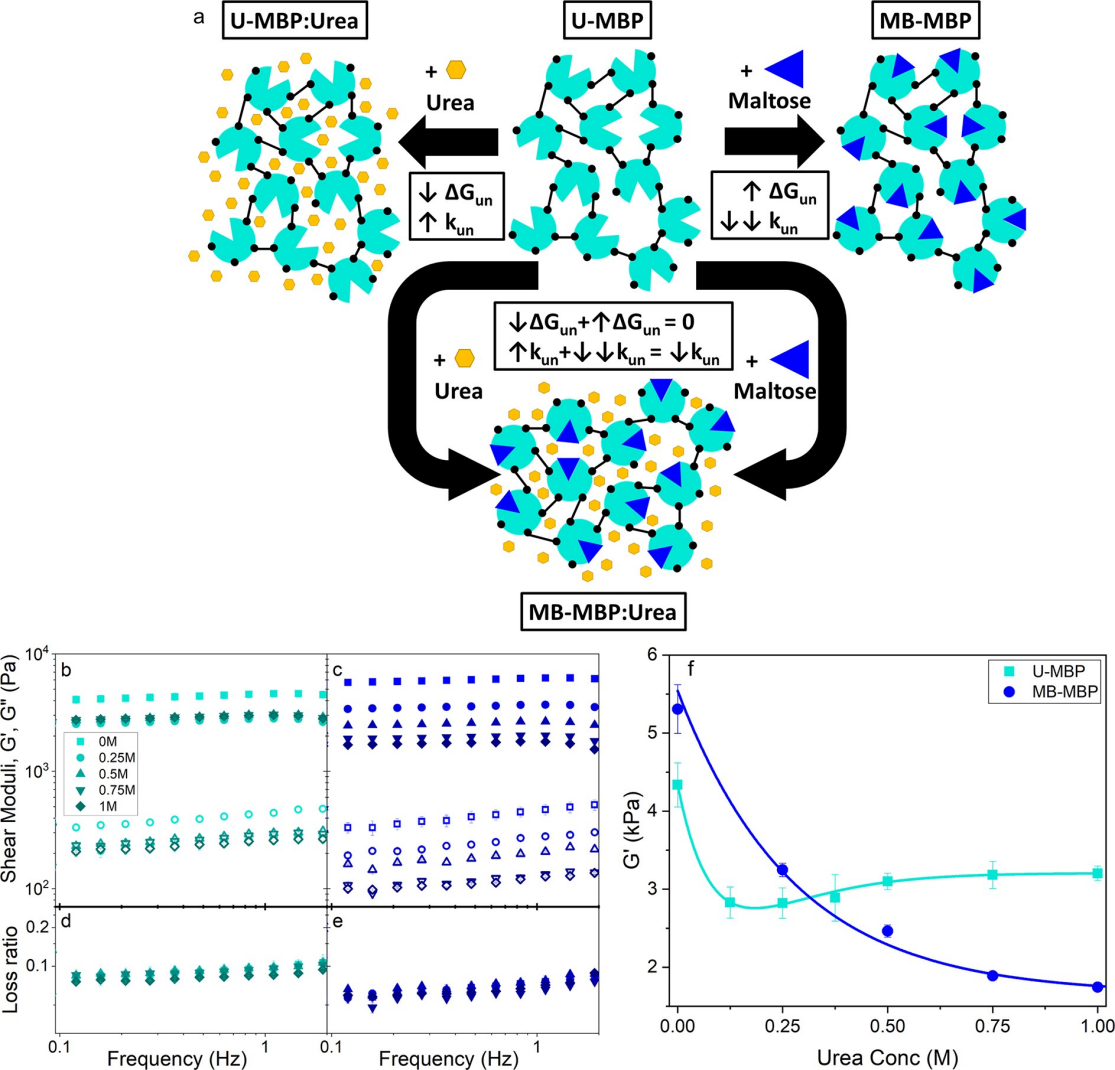
(a) Schematic representation of our model MBP hydrogel system, showing the effect on the thermodynamic stabilty, Δ*G*_UN_, and the unfolding rate, *k*_un_, upon the addition of the ligand maltose or the denaturant urea or both together. (b, c) Frequency sweeps showing the (filled) storage, *G*′, and (open) loss moduli, *G*″, of chemically cross-linked MBP hydrogels (final concentrations: 100 mg·mL^–1^ MBP, 30 mM NaPS, 100 μM Ru(II)bpy_3_^2+^) in the (b) absence (left) and (c) presence of maltose, as a function of urea concentration, where the number of repeats per urea concentration, *N* = 3. Bottom panels show the loss ratio of MBP hydrogels in the (d) absence and (e) presence of maltose. (f) Storage shear moduli of cross-linked U- and MB-MBP hydrogels extracted from the frequency sweep data (b and c) at a frequency of 1 Hz as a function of urea concentration.

Panels b and c of Figure 2 show the variation with frequency of the storage, *G*′, and loss, *G*″, moduli, which are the real and imaginary components of the complex shear modulus and describe the elasticity and viscosity of MBP hydrogels, respectively. Both moduli decrease linearly below 1 Hz in all samples as the measurement frequency is decreased. The loss ratio for all samples is below 0.1 at all measured frequencies and decreases as frequency approaches zero, demonstrating that the elastic behavior of the MBP hydrogels is dominant over the viscous behavior at all relevant time scales. The loss ratio has previously been observed as a marker for the level of unfolded protein in folded protein hydrogels. This is due to the unfolded protein chains behaving like wormlike chains losing energy upon deformation due to chain rearrangements,^13^ leading to an increase in the viscous behavior of the system; i.e., a higher proportion of unfolded protein leads to an increased *G*′′ and subsequently a larger loss ratio. The loss ratio is slightly lower from MB-MBP hydrogels compared to U-MBP hydrogels indicating an increase in the dominance of elastic behavior. Similar behavior has been observed in other protein hydrogels constructed from bovine serum albumin (BSA), where this was attributed to an increased amount of folded protein in the system.^30^ Fitting the linear section of the frequency sweeps in Figures 2b,c allows for the extraction of the storage modulus at a frequency of 1 Hz.

Figure 2f shows the dependence of *G*′ extracted at 1 Hz of U-MBP and MB-MBP hydrogels with urea concentration. From the graph we can see that both U-MBP and MB-MBP hydrogels in the presence of urea are mechanically weaker than those in the absence of urea. In addition to this reduction in mechanical rigidity, the presence of urea also reduces the thermodynamic stability of the MBP building block (Figure 1c), demonstrating that the protein thermodynamic stability directly translates to the mechanical rigidity of a protein network.

Interestingly, while the presence of urea decreases the storage modulus of both U-MBP and MB-MBP hydrogels, at high urea concentrations (>0.5M) MB-MBP hydrogels are mechanically weaker than those constructed from U-MBP. Furthermore, the different gels exhibit different trends with urea concentration. U-MBP hydrogels show an initial decrease in *G*′ with urea concentration before the trend inflects at ∼0.25 M urea, after which *G*′ slightly increases to a plateau value of ∼3 kPa. This inflection is not observed in MB-MBP hydrogels, which display a continuous decrease appearing to plateau below ∼2 kPa. The comparatively weaker MB-MBP gels at high urea concentrations suggests that increasing the kinetic stability of MBP, via binding maltose, results in weaker hydrogels. Additionally, increasing the kinetic stability of MBP removes the inflection in the urea dependency of *G*′ observed in the U-MBP hydrogels (Figure 2f). These trends with urea concentrations were fitted with exponential fits to investigate the trends with urea concentration observed in U-MBP and MB-MBP hydrogels. For MB-MBP only a single exponential decay function was necessary to fit the data, suggesting that only a single mechanism was responsible for the change in *G*′, namely that the thermodynamic stability of the building block directly governs the *G*′ of the network. A dual exponential decay function was necessary to adequately fit the U-MBP data (Figure S3), suggesting that there is an inflection in the data and that there are two competing mechanisms defining the urea dependency, the first being the direct dependency on the thermodynamic stability (seen in MB-MBP hydrogels), while the second mechanism is unclear. This additional underlying mechanism may be due to perturbation of the hydrogel formation process. To investigate this possibility, we analyze the gelation curves of MBP hydrogels.

### Perturbation of Hydrogel Network Formation

**Figure 3:**
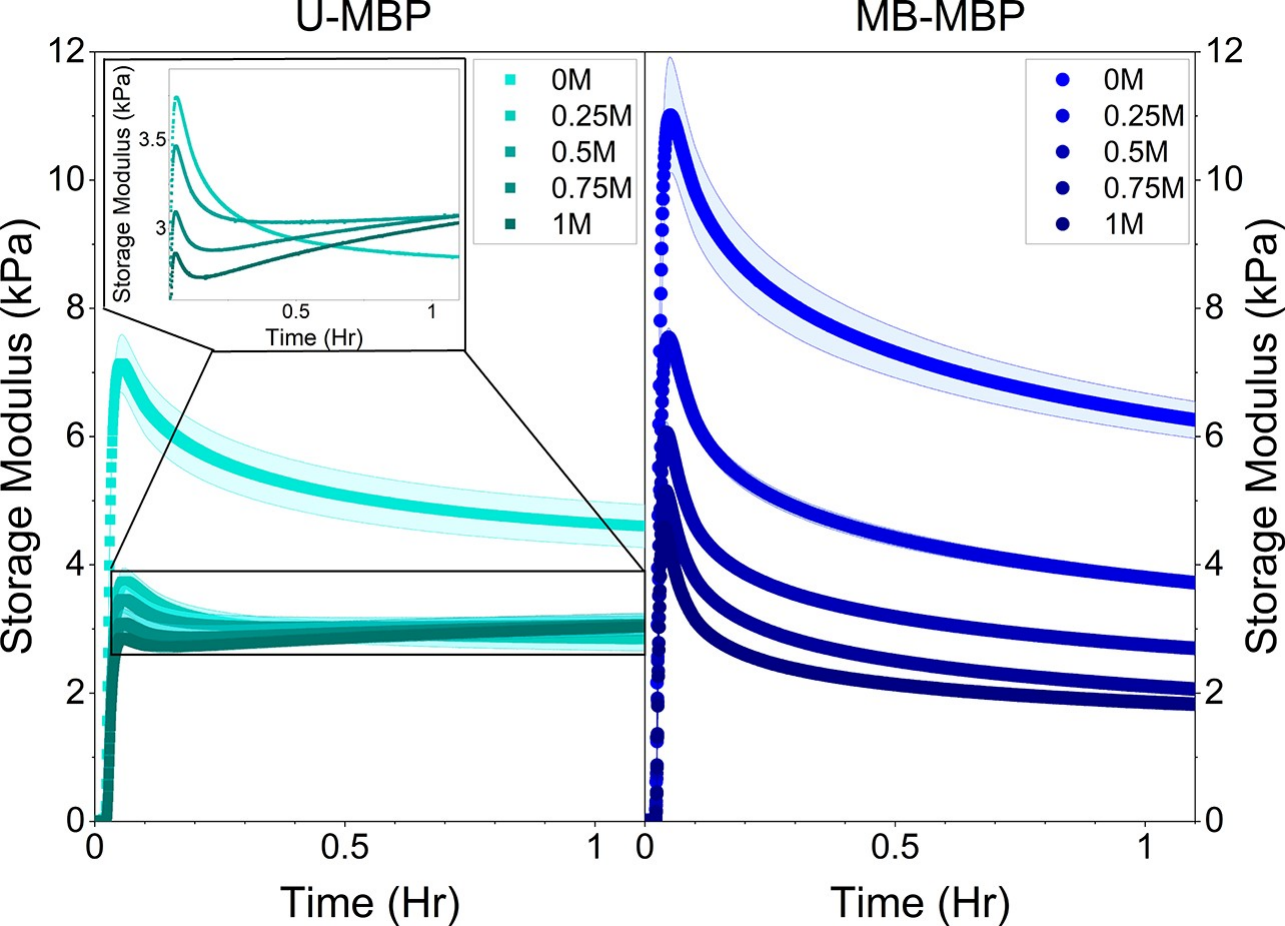
Gelation curves of MBP hydrogels (100 mg·mL^–1^ MBP, 30 mM NaPS, 100 μM Ru(II)bpy_3_^2+^) depicting the evolution of *G*′ as a function of time in the absence (left) and presence (right) of maltose at various urea concentrations (increasing urea concentration indicated by darker shading). *G*′ values were recorded every 3 s at 0.5% strain and a frequency of 1 Hz where the number of repeats per urea concentration, *N* = 3.

The gelation curves (Figure 3a) show the evolution of the storage modulus (*G*′) during the formation of MBP hydrogels at various urea concentrations in the absence and presence of maltose.

Without the addition of urea, the gelation curves show the expected profile of an initial sharp increase in *G*′ due to photoactivated chemical cross-linking, followed by slow relaxation down to a plateau value. Greater values of *G*′ are observed in the presence of maltose, in agreement with our previous work on MBP hydrogels.^28^ The end point *G*′ values are consistent with those observed from frequency sweep measurements (Figure 2f). MB-MBP hydrogels exhibit diminished *G*′ values with increasing urea concentration, but no significant alteration to the profile of the gelation curve is observed regardless of urea concentration. In contrast, the gelation curves of U-MBP hydrogels deviate from the standard profile (i.e., initial sharp increase followed by slow relaxation) to a different profile as the concentration of urea is increased. The change in the U-MBP gelation profile relative to 0 M urea is greatest at 1 M urea. This profile can be described as a sharp increase followed by a more rapid relaxation and ending with a slow increase up to the final plateau value of *G*′ (Figure 3). The change in the relaxation behavior of U-MBP hydrogels, from reducing *G*′ with time to increasing *G*′ with time, is indicative of additional physical cross-links forming over time. Importantly, this alteration to the gelation profile due to the addition of urea (decrease in thermodynamic stability) is only observed in U-MBP, suggesting that the diminished kinetic stability of the protein plays a key role in defining the formation and relaxation kinetics of folded protein hydrogels.

### Alteration of Hydrogel Relaxation Mechanism

As discussed above, U-MBP hydrogels show significant perturbations to their postchemical cross-linking relaxation as the concentration of urea is increased. We would expect this perturbation if the relaxation mechanism were altered from being dominated by the unfolding of protein domains^28^ to being dominated by the entanglement of unfolded protein, with additional physical cross-links forming over time.^29^ Here, we define entanglement as referring to cohesive entanglement which arises due to interchain cohesion with short parallel alignments of neighboring segments physically cross-linked together,^51−53^ as opposed to topological entanglement^54,55^ (physical restrictions on lateral chain movement due to other chains in 3-D space). The process of polymer entanglement, both cohesive^56,57^ and topological,^58^ is important in biological systems, such as intrinsically disordered proteins^59−61^ and DNA supercoiling,^58,62,63^ and has recently been studied using structural techniques^64^ combined with in-depth computational modeling.^65^ In order to investigate the possibility and importance of entanglement in our hydrogels, we analyze the complex kinetic profiles shown in Figure 3 in more detail by fitting a previously used empirical equation^28,30^ (eq 1).
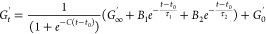
1

Fitting the gelation curves with this function allows the extraction of several key parameters related to the relaxation behavior including the time constants of the relaxation modes, τ_1_ and τ_2_, and the relaxation coefficients of these modes, *B*_1_ and *B*_2_. In previous work,^28^ we have attributed the shorter, first relaxation mode to the rearrangement of the network immediately postphotochemical cross-linking, where due to the rapid photochemical cross-linking of the protein into a hydrogel network the system is in a frustrated state with high internal stresses that are relaxed by internal rearrangement of the network. Furthermore, we have demonstrated (in 0 M urea) that the second slower relaxation mode is due to the unfolding of the protein building block; i.e., the internal stresses of the network are further relaxed by the protein domains unfolding removing the robust spring like folded structure and providing “slack” in the system in the form of unfolded protein chains.

**Figure 4:**
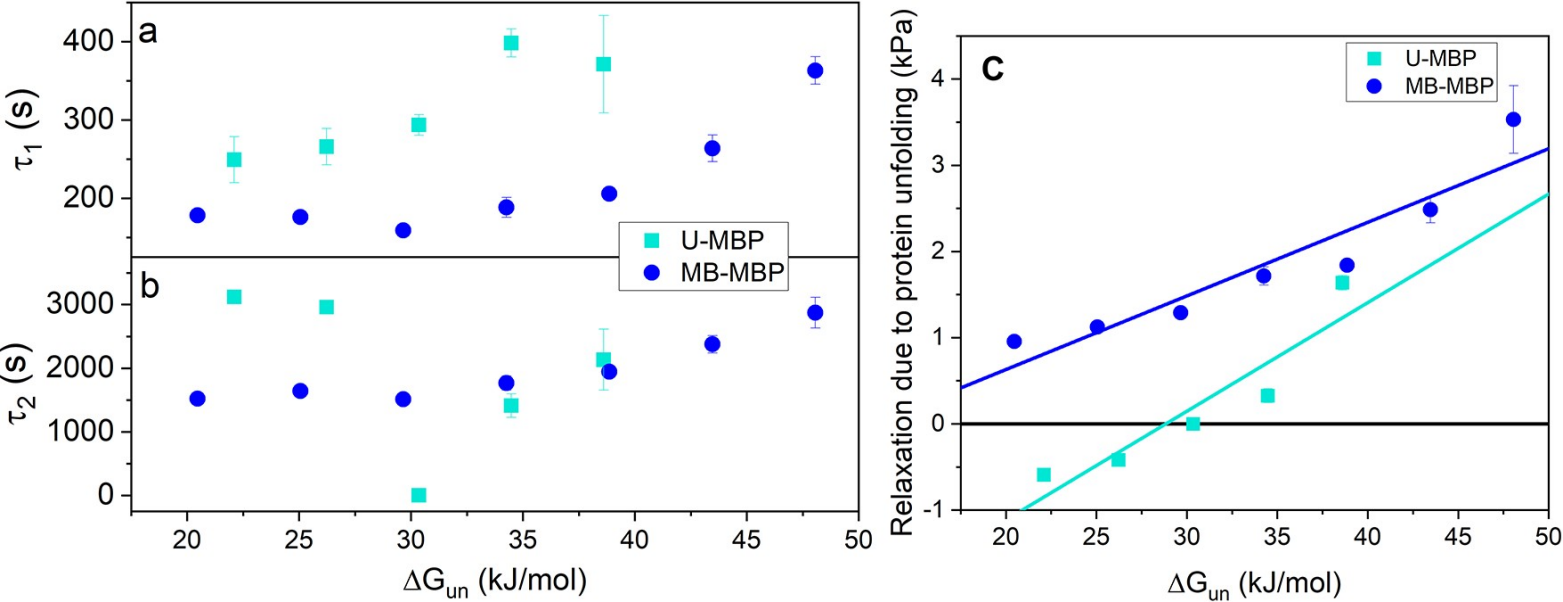
Relaxation time constants of the (a) first, τ_1_, and (b) second relaxation mode, τ_2_, extracted from the gelation curves as a function of MBP thermodynamic stability, in the absence (light blue) and presence (dark blue) of maltose. (c) MBP thermodynamic stability dependency of the relaxation coefficient of the second relaxation mode, B_2_, from eq 1, which characterizes the reduction in storage modulus as the MBP building block unfolds postphotochemical gelation. Where the number of repeats per urea concentration, *N* = 3.

Combining the results extracted from the gelation curves (Figure 3a,b) with our DSC measurements (Figure 1c) yields figure 4. To ensure adequate comparison over the Δ*G*_un_ range, we have measured MB-MBP hydrogels at additional urea concentrations of 1.25 and 1.5 M which correspond to Δ*G*_un_ of ∼ 25 and ∼ 20 kJ·mol^–1^, respectively. Figure 4a shows how these extracted relaxation time constants, τ_1_ and τ_2_, vary as a function of the thermodynamic stability of both U-MBP and MB-MBP. Both hydrogels (U-MBP and MB-MBP) show a reduction in the τ_1_ relaxation with a decrease in MBP thermodynamic stability. This reduction in τ_1_ suggests faster network rearrangement, which may be due to an overall softer network constructed from less thermodynamically stable MBP, allowing for easier relaxation of the network. This interpretation is consistent with the shear moduli values observed in figure 2f; i.e., weaker hydrogels exhibit faster network rearrangement timescales.

Similarly, for MB-MBP hydrogels τ_2_ is reduced as the thermodynamic stability of MBP is reduced (concentration of urea is increased). This suggests that the MBP building block unfolds more rapidly as the thermodynamic stability of the building block is lowered. In stark contrast, the τ_2_ relaxation for U-MBP hydrogels has a much more complex Δ*G*_un_ dependency. Initially, a reduction in τ_2_ with decreasing Δ*G*_un_ is observed, with τ_2_ values in good agreement with those observed from MB-MBP hydrogels (i.e., at Δ*G*_un_ ≈ 38 kJ·mol^–1^ τ_2_ = 2100 ± 500 s (U-MBP) and 1900 ± 100 s (MB-MBP) and at Δ*G*_un_ ≈ 34 kJ·mol^–1^ τ_2_ = 1400 ± 200 s (U-MBP) and 1700 ± 100 s (MB-MBP)). This consistency in the measured τ_2_ timescales indicates that the thermodynamic stability of the protein building block governs the unfolding rate of the protein building block. However, a different behavior is measured for τ_2_ when Δ*G*_un_ < 32.5 kJ·mol^–1^ for U-MBP and MB-MBP hydrogels: for U-MBP hydrogels as Δ*G*_un_ continues to decrease a large drop down to τ_2_ ≈ 5 s at Δ*G*_un_ = 30.4 kJ·mol^–1^ is observed followed by a sharp increase up to ∼3000 s as Δ*G*_un_ is further reduced. The inflection in Δ*G*_un_ dependency of τ_2_ of U-MBP hydrogel is reminiscent of the inflection in the urea concentration dependency of *G*′. This suggests that the origin of the difference in the *G*′ trends between U-MBP and MB-MBP hydrogels is the postchemical cross-linking relaxation of these hydrogels.

As mentioned above, a possible explanation for the differing behavior observed between U-MBP and MB-MBP hydrogels is a regime change in the relaxation behavior from an unfolding dominated relaxation to an entanglement dominated relaxation. The second relaxation coefficient B_2_ gives a measure of the amount of protein unfolding during relaxation. Surprisingly, Figure 4c shows a reduction in B_2_ as a function of U-MBP and MB-MBP thermodynamic stability, suggesting less protein is unfolded within the gels postgelation. However, previous CD measurements^28^ and an invariance of the efficiency extracted from stress–strain curves (Figures S4 and S5) of U-MBP and MB-MBP hydrogels across urea concentrations suggest that the level of unfolded protein at the end of the time course is consistent across all gels. This implies that the measured reduction in B_2_ is the result of more protein unfolding during photo-chemical cross-linking as opposed to during network relaxation. Interestingly in U-MBP hydrogels the value for B_2_ changes parity (from positive to negative) at Δ*G*_un_∼30 kJ·mol^–1^, coincident with the drop in τ_2_ (Figure 4b). This switch in parity suggests two possibilities: (i) protein domains are refolding during relaxation resulting in an increase in *G*′ due to additional rigid folded protein in the system or (ii) the rapid unfolding of protein during initial photo-chemical cross-linking leads to entanglement of unfolded protein chains occurring during relaxation, which would result in an increase in *G*′ due to additional physical cross-links in the system. The former of these explanations seems unlikely given that the loss ratio of U-MBP and MB-MBP (Figure 2d) does not significantly vary with increasing urea concentration suggesting that the amount of unfolded protein is consistent across all gels.^28^ To further confirm that the level of folded protein is consistent across all urea concentrations, we perform load–unload measurements (Figure S4) to determine the energy dissipated in the gels and subsequently calculate the efficiency of the hydrogels (Figure S5). The load–unload efficiency of folded protein hydrogels has been shown to be correlated to the amount of unfolded protein.^25,30^ At all urea concentration the efficiency of U-MBP hydrogels is shown to be consistent, adding further evidence that the proportion of folded protein remains the same and, hence, making it very unlikely that significant refolding is occurring. Furthermore, the U-MBP is least thermodynamically stable at these points, making it more unlikely to spontaneously refold. Taken together, this suggests that as the thermodynamic stability of the kinetically less stable U-MBP is reduced the relaxation behavior undergoes a regime change from an unfolding dominated regime to an entanglement dominated one.

We would expect hydrogels that exhibit entanglement dominated relaxation to exhibit different nonlinear characteristics at high strains. To investigate this, we perform strain amplitude sweep experiments on U-MBP and MB-MBP hydrogels at varying urea concentrations (Figure S6). These measurements showed two key results: (i) that the strain-stiffening profile of U-MBP was significantly altered by the presence of urea whereas MB-MBP maintained a similar shape profile at all urea concentrations and (ii) that the breaking strain of U-MBP decreases as the concentration of urea is increased (note such a reduction is not observed in MB-MBP hydrogels). We would expect these results if there was significant cohesive entanglement points in the hydrogels, as these points would restrict the extensibility of the network compared to samples which show a lower degree of entanglement. These measurements suggest that cohesive entanglements are present in U-MBP hydrogels at high urea concentrations (>0.5 M) and that they are mechanically dominant at high strains (>50%). Additionally, we performed small-angle X-ray scattering (SAXS) experiments on the hydrogels (Figure S7a,b) to investigate the change in hydrogel architecture and confirm the occurrence of entanglements. Recent computational work on colloidal based networks has shown that such networks consist of fractal-like clusters.^66^ The interconnection and 3D arrangement of these clusters were shown to form a rigid network dominating the mechanical behavior;^67^ this has also been validated experimentally.^68^ Furthermore, from previous characterization of protein hydrogels we expect the structure to consist of fractal-like clusters of cross-linked protein connected by an intercluster region.^28,30,69−71^ Extracting the fractal dimension, *D*_f_ (Figure S7c), of these clusters (which can be thought of as a measure of the density of the cluster) from the SAXS curves shows an increase in *D*_f_ for U-MBP hydrogels with decreasing thermodynamic stability, suggesting denser clusters consistent with what we would expect from an entanglement dominated system. No change in *D*_f_ is observed for MB-MBP hydrogels.

Our results show that while a reduction in the thermodynamic stability of MBP drives more rapid unfolding of the protein building block, it is the kinetic stability of MBP that limits and defines the relaxation behavior. Furthermore, hydrogels constructed from less kinetically stable protein undergo a regime change in their relaxation behavior from unfolding-dominated to entanglement-dominated as the protein thermodynamic stability is lowered.

## Conclusions

In this work, by characterizing the mechanics of U-MBP and MB-MBP hydrogels in urea, we demonstrate that the distinct thermodynamic and kinetic stability of the protein building block on the molecular level have important roles in governing the bulk mechanical rigidity, formation, and relaxation kinetics of folded protein networks.

The thermodynamic stability of the protein building block directly translates to the bulk mechanical properties of the network, i.e., the more thermodynamically stable the protein building block the more mechanically rigid the resultant gel. The kinetic stability, on the other hand, governs the formation and relaxation process of the hydrogel network, which subsequently affects the bulk mechanical rigidity of the network.

Previously, we have shown that simultaneously increasing the thermodynamic, kinetic, and mechanical stability of MBP hydrogels via the addition of maltose results in a 62% increase in the storage modulus of MBP hydrogels.^28^ However, in this previous study we were unable to dissect the importance of each stability in governing the observed increase in storage modulus. Our results in this work show that the storage modulus of MBP hydrogels upon the binding of maltose is dominated by the increase in protein thermodynamic stability.

The formation and relaxation kinetics of folded protein hydrogels were also examined. A reduction in the MBP thermodynamic stability resulted in a decreased protein unfolding relaxation time constant, thereby driving faster relaxation mechanics. However, it was shown that the kinetic stability is the limiting factor in defining the relaxation mechanism and the less kinetically stable protein undergo a regime change from an unfolding-dominated relaxation to an entanglement-dominated one. This regime change is characterized by a reversal in the relaxation behavior; i.e., *G*′ increases over time as opposed to decreases. Additionally, this regime significantly alters the nonlinear shear stiffening profile of the hydrogels. These results suggest that folded protein hydrogels have a two-regime relaxation behavior that can be accessed by tuning both the thermodynamic and kinetic stability of the protein building block. While rheological characterization and small-angle scattering have aided in identifying a regime shift in MBP hydrogels, it was not possible to disentangle the mechanical contribution of the photochemical cross-links and the cohesively entangled physical cross-links. To disentangle the specific contribution of these two types of cross-linking a new model system would need to be developed, such a system would take inspiration from the work of Olsen et al.^72,73^ In their work Olsen et al. used a bespoke protein construct containing coiled-coil regions and unstructured peptide regions ending in a disulphide forming cysteine, which were then made into long polyconstructs via the formation of disulphide bonds. The group were able to deconvolute the mechanical contributions of the coiled-coil interactions and entanglements to the modulus of their hydrogels, via the addition of reducing agents to break the disulphide bonds removing the long chains and hence the entanglements.^73^ Their work gives a blueprint for how the importance of chemical cross-links and entanglements could be deconvoluted in folded protein hydrogels.

**Figure 5:**
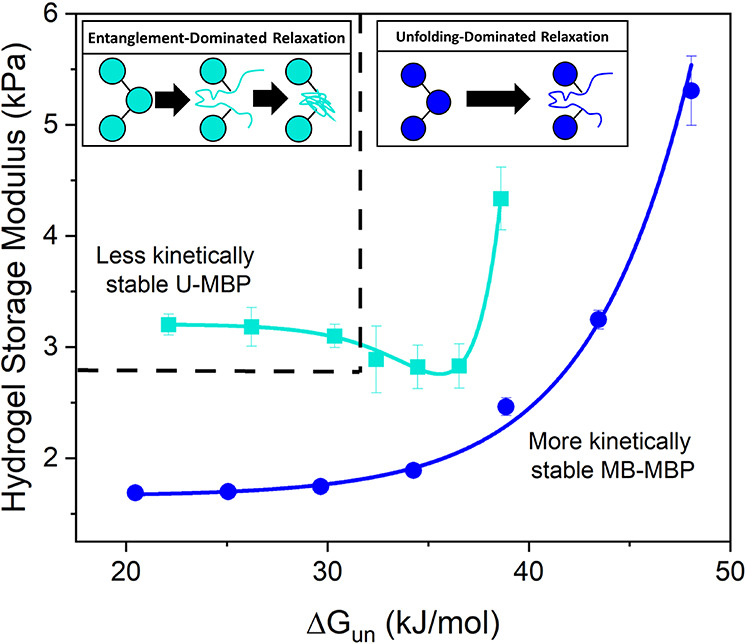
Storage shear moduli of cross-linked MBP hydrogels extracted from the frequency sweep data (Figure 2b,c) at a frequency of 1 Hz as a function of unbound (open symbols) and maltose-bound (closed symbols) MBP thermodynamic stability in the absence and presence of 10 mM maltose, respectively.

In this work, we have shown that MBP hydrogels in the entanglement relaxation regime exhibit both enhanced rigidity compared to hydrogels constructed from similar thermodynamic stable building blocks (Figure 5) and load–unload energy dissipation that is independent of the thermodynamic stability of the building block (Figure S5). This has exposed control of the regime shift from unfolding to entanglement dominated relaxation as a route to control folded protein hydrogel properties, showing the importance of unfolded protein entanglement in hydrogel mechanics. Others have similarly found that there are advantages to allowing or including entanglement in hydrogels, for example, Suo et al., who demonstrated the mechanical importance of physical entanglements within polymer hydrogels, finding that highly entangled hydrogels are ideal load bearing materials.^74^ Both Suo et al.’s work and our study demonstrate the importance and usefulness of entanglement within networks for the development of biomaterials. For example, polymer entanglement has been used to drive the adhesion of a double network hydrogel wound dressing triggering effective skin wound healing in vivo with lower immune response compared to commercial tissue adhesives.^75^ Such materials provide crucial insight and allow for development of dynamic entanglement-based hydrogels for biomedical applications.

The development of innovative biomaterials offers enormous potential for addressing significant challenges in medical and healthcare technologies. As life expectancy increases, pioneering methods are needed to replace and restore tissues and organs in the body, to improve tissue engineering, and to develop robust and responsive drug delivery approaches.^18,76−80^ By understanding protein stability at the molecular level, we demonstrate the differential roles of these stabilities in defining protein network mechanics and reveal a method to tailor assemblies of folded proteins for specific applications.

## Materials and Methods

### Materials

Tris(2,2′-bipyridyl)dichlororuthenium(II) hexahydrate (Ru(BiPy)_3_), sodium persulfate (NaPS), 1,4-dithiothreitol (DTT), d-(+)-maltose monohydrate, sodium phosphate dibasic, and sodium phosphate monobasic were obtained from Sigma-Aldrich and used without further treatment.

### Protein Preparation

MBP with an N-terminal hexa-histidine tag was prepared using a mutated pMal-c5x vector with a stop codon inserted at position 378 by Q5 mutagenesis. The mutated vector was transformed into the expression host *E. coli* BL21 (DE3) pLysS competent cells. Selected colonies were grown overnight in Lysogeny Broth (LB) at 37 °C, 200 rpm to form starter cultures. Starter cultures (2 mL) were used to inoculate autoinduction media^81^ (0.5 L, containing a 100 μg·mL^–1^ concentration of the antibiotic carbenicillin) in 2.5 L conical flasks. Cultures were incubated for 48 h at 37 °C, 200 rpm before cells were harvested at 8000 rpm for 45 min. The harvested pellets were resuspended in lysis buffer (0.1% Triton X-100, 1 mM PMSF, 20 mM benzamidine, 20 mM Tris, 300 mM NaCl, 10 mM imidazole, pH 8), homogenized, and incubated for 1 h in the presence of DNAase I (ThermoFisher Scientific Inc.). Cell solutions were passed through a cell disruptor (30 K psi, 25 °C) to ensure complete lysis before centrifuging at 25000 rpm for 25 min to pellet the cell debris and collect the lysate.

To purify the MBP from the lysate, it was loaded onto a Ni-NTA resin column overnight at 2 mL·min^–1^ to ensure maximum binding of the hexahistidine-tagged MBP. The column was then equilibrated in wash buffer (20 mM Tris, 300 mM NaCl, 10 mM imidazole, pH 8) before the protein was eluted with elution buffer (20 mM Tris, 300 mM NaCl, 500 mM imidazole, pH 8) in a ratio of 1:3 to wash buffer. The purified protein was dialyzed into water and freeze-dried for storage at −20 °C. An average yield of MBP was 300 mg/L. Three batches of MBP were prepared in order to produce the necessary amount of protein for rheology and scattering experiments.

### Sample Preparation

As previously published,^28^ hydrogel samples are prepared by mixing in a 1:1 ratio a stock of MBP protein (200 mg·mL^–1^) and 2× cross-link reagent stock (60 mM NaPS, 200 μM Ru(BiPy)_3_, 2XM Urea) for final protein and reagent concentrations of 100 mg·mL^–1^ MBP (MBP), 30 mM NaPS, 100 μM Ru(BiPy)_3_, XM Urea. Samples were then gelated via photochemical cross-linking with the urea denaturant present during and after gelation.

### Differential Scanning Calorimetry

MBP pregelation solution (10 μL) and hydrogel samples were loaded into Tzero hermetically sealed pans, and sodium phosphate buffer (9.26 μL) was used as a reference. Samples were heated from 30 to 95 °C at a rate of 10 °C·min^–1^. In these measurements, the samples contained varying concentrations of urea and either 0 or 10 mM maltose. Measurements were performed on a TA Instruments Q2000 DSC, which were calibrated with a sapphire standard of known heat capacity to ensure accurate determination of the protein heat capacity.

### Rheometry

Mechanical characterization experiments of MBP hydrogel samples were performed on an Anton Paar MCR 502 stress-controlled rheometer (Anton Paar GmbH, Austria) in parallel plate configuration (with a plate diameter of 8 mm). Photochemical cross-linking was initiated and controlled via illumination by blue LED (peak emission at 452nm) at a current of 0.48A. To prevent evaporation, during this process low viscosity silicone oil (approximately 5 ct) was placed around the geometry. The silicone oil should present no systematic error on rheometric data as this is below the rheometer’s torque range. Time sweep gelation measurements were conducted at a frequency and shear strain of 1 Hz and 0.5%, respectively. Gelation curves were fitted with an empirical function (eq 1) where *C* and *t*_0_ are the rate and midpoint of increase of *G*′ due to photochemical cross-linking respectively, *B*_1_ and *B*_2_ are the coefficients of the first and second relaxation mode respectively,  is the plateau value of the storage modulus postgelation and postrelaxation, and finally *G*′_0_ is the storage modulus of the sample pregelation. All other terms are defined within the main text. This function has previously been used to fit gelation curves of folded protein hydrogels assembled using this photochemical cross-linking method.^28^

### Small Angle X-ray Scattering (SAXS)

SAXS measurements were conducted in the Materials Characterization Laboratory of the ISIS Neutron and Muon Source on the Nano-inXider instrument (Xenocs, Sassenage, France) using a microfocus sealed-tube Cu 30 W/30 μm X-ray source (Cu K-α, λ = 1.54 Å). Samples and buffer were loaded in 1 mm path length glass capillary tubes. The *q*-range investigated was 0.0045–0.37 Å^–1^, and measurements were made at room temperature. Raw SAXS data from the Nano-inXider was normalized and radially averaged using Foxtrot (SOLEIL Software) to produce 1D scattering curves. SAXS curves were fitted using SasView (http://www.sasview.org) in accordance with eq 2
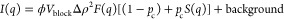
2
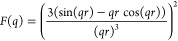
3
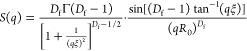
4where *F*(Q) is an ellipsoidal form factor^82^ and *S*(Q) is a fractal structure factor to model the geometry of the clustering of objects of the form *F*(Q).^83^*D*_f_, ξ, and *R*_0_ are defined as the mass fractal dimension, correlation length, and minimum cutoff length-scale defined by the ellipsoid form factor, respectively.

## Supporting Information Available

(Figure S1) Exemplar DSC curve of MBP melting. (Figure S2) SAXS curves of uncross-linked U- and MB-MBP. (Figure S3) Additional fit models to urea dependence of U-MBP hydrogels *G*′. (Figure S4) Stress–Strain curves of MBP hydrogels. (Figure S5) Extracted energy dissipation and efficiency of MBP hydrogels as a function of urea concentration. (Figure S6) Strain amplitude ramps of MBP hydrogels as a function of urea concentration. (Figure S7) SAXS curves of cross-linked U- and MB-MBP hydrogels and extracted fractal dimensions as a function of urea concentration (PDF)

## Notes

The data has been deposited and can be found here: https://doi.org/10.5518/1174.
