# Supplementary material for: Tuning Protein Hydrogel Mechanics through Modulation of Nanoscale Unfolding and Entanglement in Postgelation Relaxation
Source: ACS Nano. 2022 Jun 22;16(7):10667–78. doi: 10.1021/acsnano.2c02369 (PMC9331141; doi:10.1021/acsnano.2c02369)
Supplement: Supplementary file 1 — nn2c02369_si_001.pdf [file nn2c02369_si_001.pdf]

Supporting Information

Tuning protein hydrogel mechanics through modulation of nanoscale unfolding and entanglement in post-gelation relaxation

Matt D G Hughes[1], Sophie Cussons[2,3], Najet Mahmoudi[4], David J Brockwell[2,3], Lorna Dougan[*,1,2]

1 School of Physics and Astronomy, Faculty of Engineering and Physical Sciences, University of Leeds, Leeds, LS2 9JT, UK

2 Astbury Centre for Structural Molecular Biology, University of Leeds, Leeds, LS2 9JT, UK

3 School of Molecular and Cellular Biology, Faculty of Biological Sciences, University of Leeds, Leeds, LS2 9JT, UK

4 ISIS Neutron and Muon Spallation Source, STFC Rutherford Appleton Laboratory, Oxfordshire, OX11 0QX, UK

*Corresponding Author: Lorna Dougan, L.Dougan@leeds.ac.uk

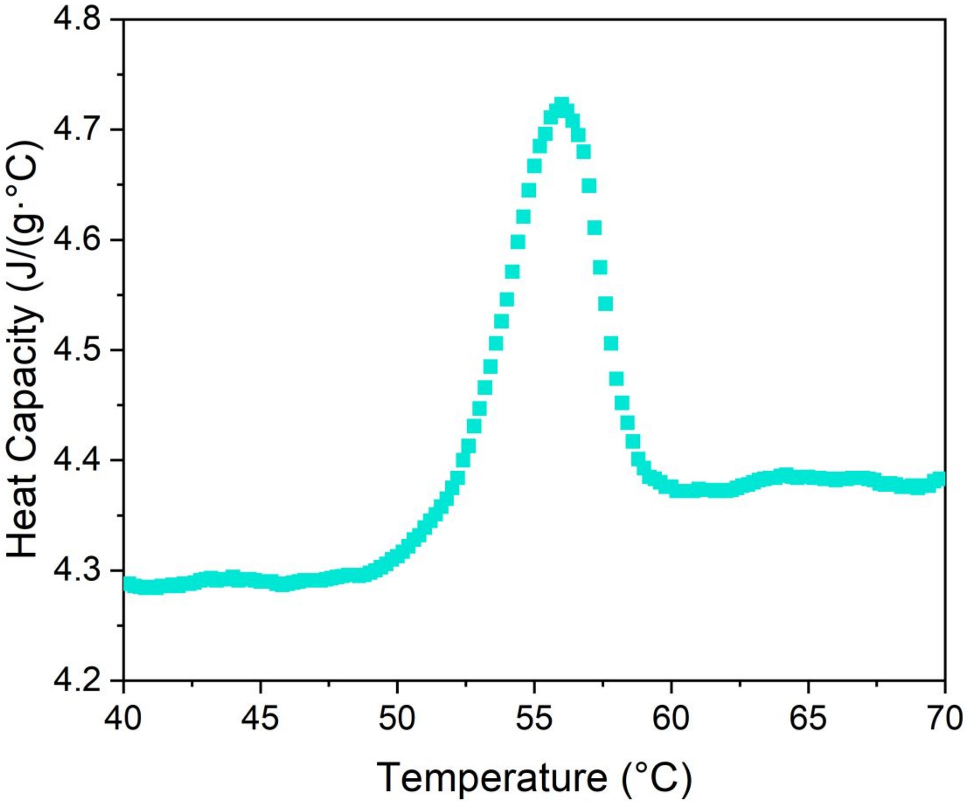

Figure S1: Exemplar DSC curve of U-MBP at 100mg/ml, showing the evolution of heat capacity as a function of increasing temperature. The temperature was increased at a rate of 10°C/min.

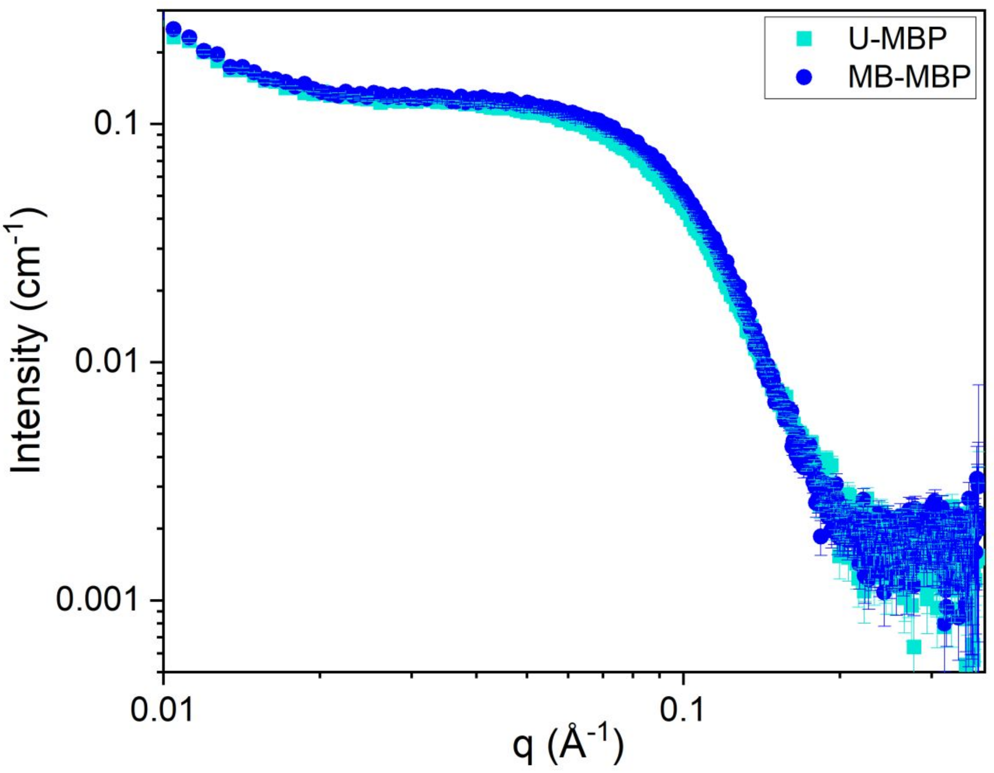

Figure S2: SAXS curves of 100mg/ml BSA solutions in the absence (light blue) and presence (dark blue) of 10mM maltose.

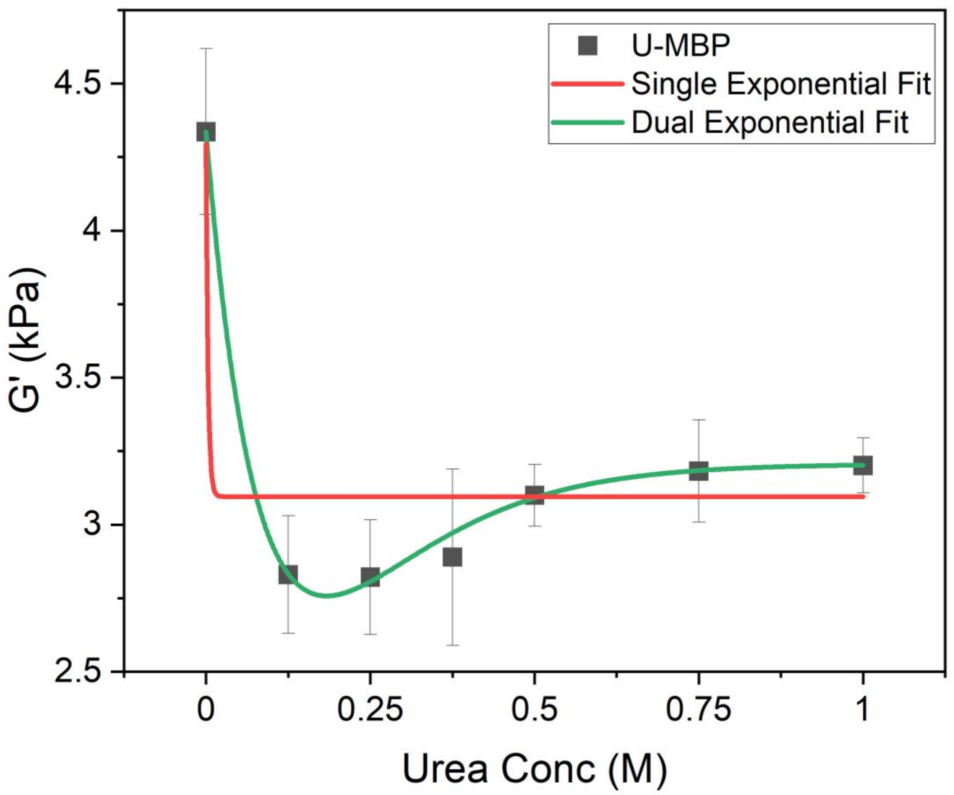

Figure S3: The urea concentration dependence of the storage modulus of U-MBP hydrogels. Two potential fits to the data are shown: (red) single exponential decay to model single dependency mechanism, (green) dual exponential decay to model two differing dependency mechanisms.

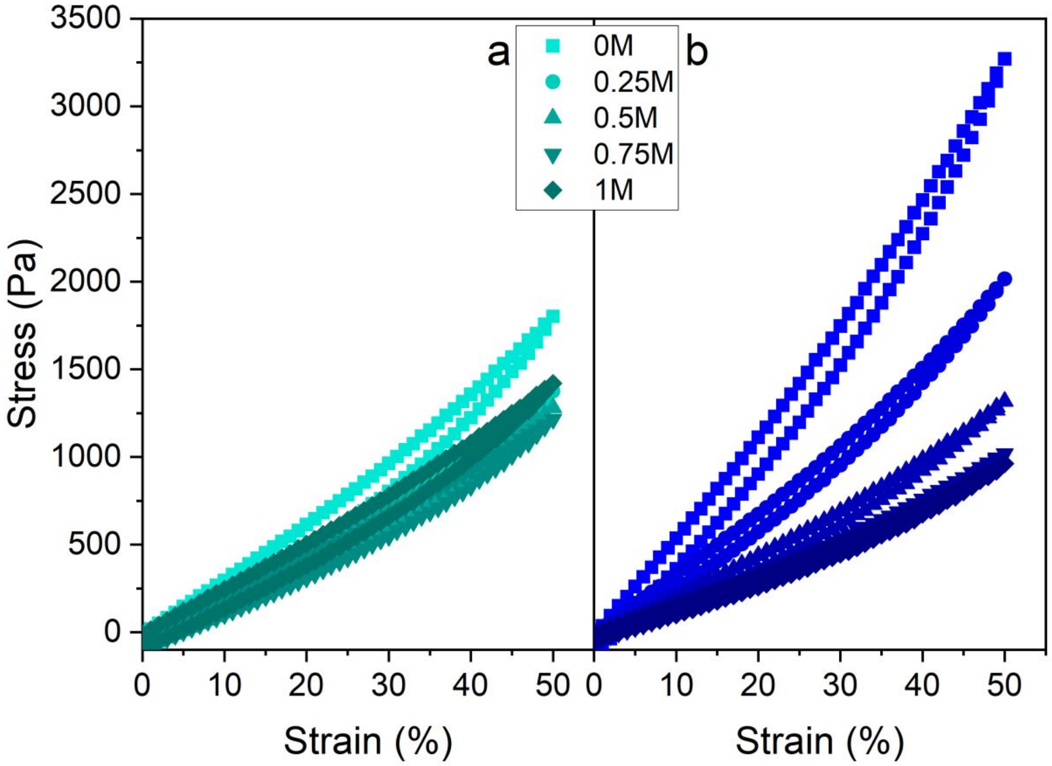

Figure S4: a) Stress-strain curves of chemically cross-linked MBP hydrogels (final concentrations: 100mg/ml MBP, 30mM NaPS, 100μM Ru(II)bpy$_3^{2+}$) in the **a)** absence and **b)** presence of maltose as a function of urea concentration. Samples were strained to 50% at a rate of 1%/s and then unloaded down to 0% at the same rate.

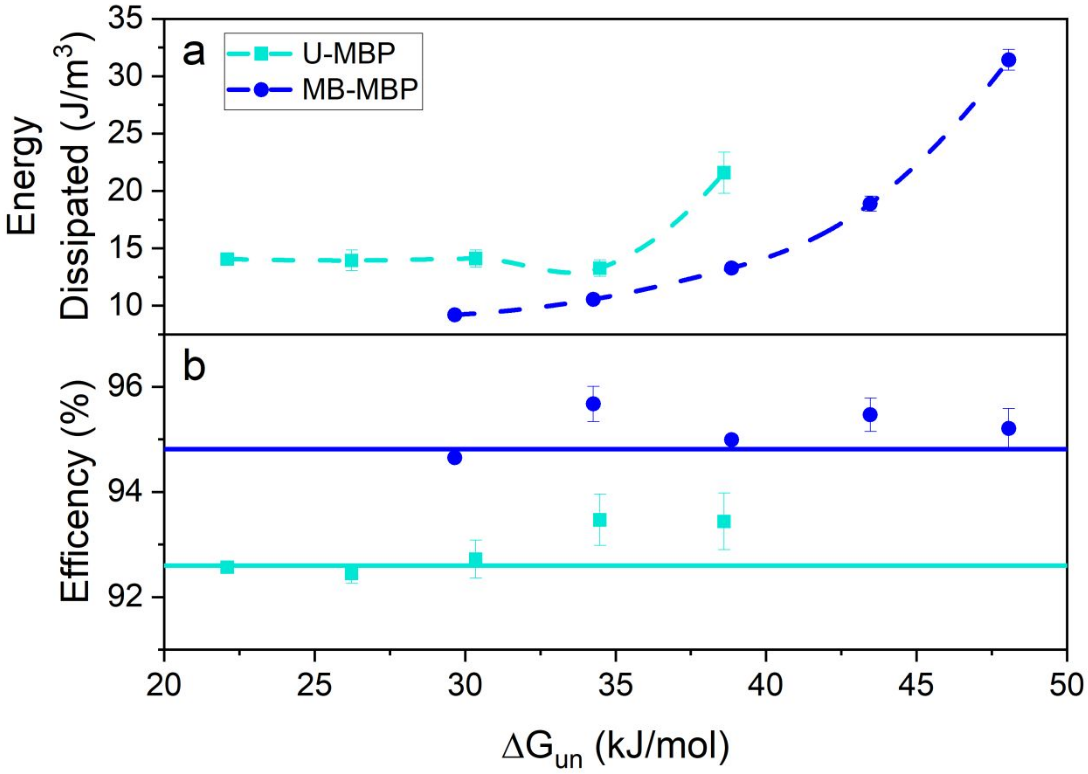

Figure S5: **a)** Energy dissipation and **b)** efficiency during load-unload cycle of MBP hydrogels in the absence (light blue) and presence (dark blue) of maltose as a function of MBP thermodynamic stability. Solid lines represent average fits to the efficiency data in the absence and presence of maltose. Dashed lines shown as a guide for the eye.

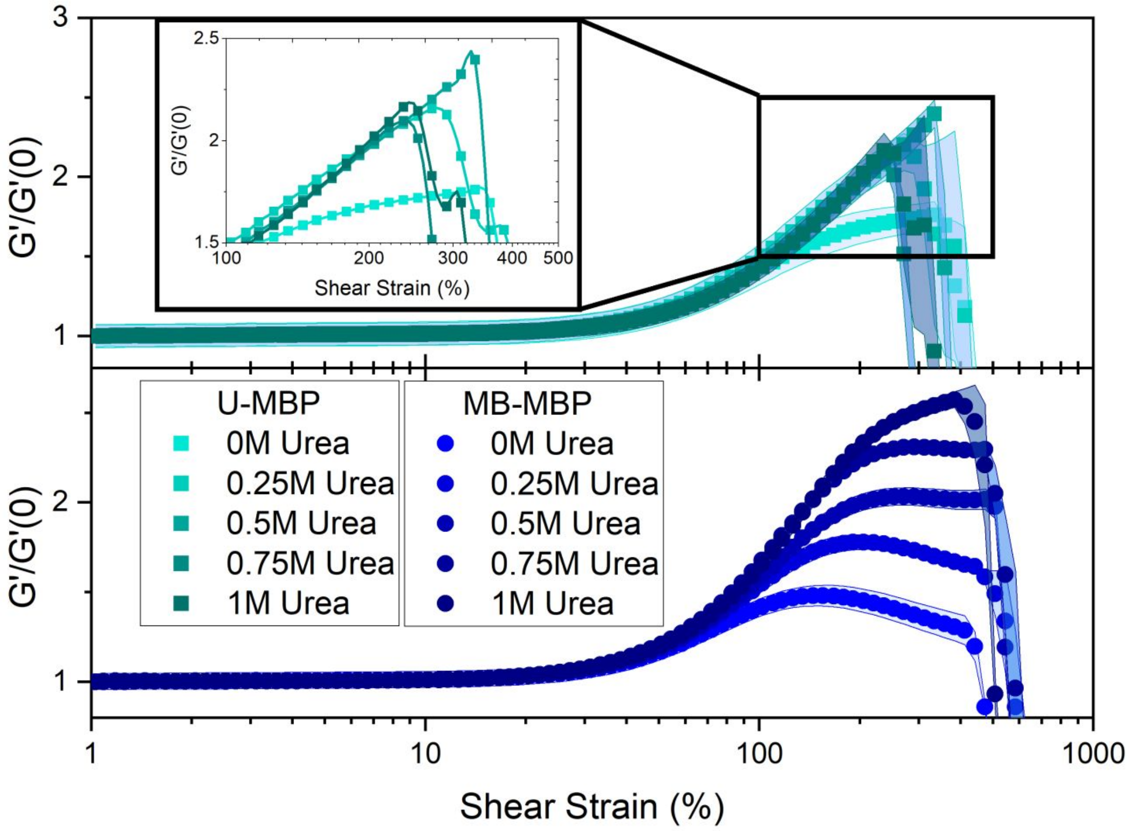

Figure S6: Storage and loss moduli of (top) U-MBP and (bottom) MB-MBP hydrogel as a function of applied oscillation strain at 1 Hz. (inset) Enlargement of the strain-stiffening regime, plotted without error bars for clarity. Error bars and ribbons show the standard errors, where number of repeats, N = 3.

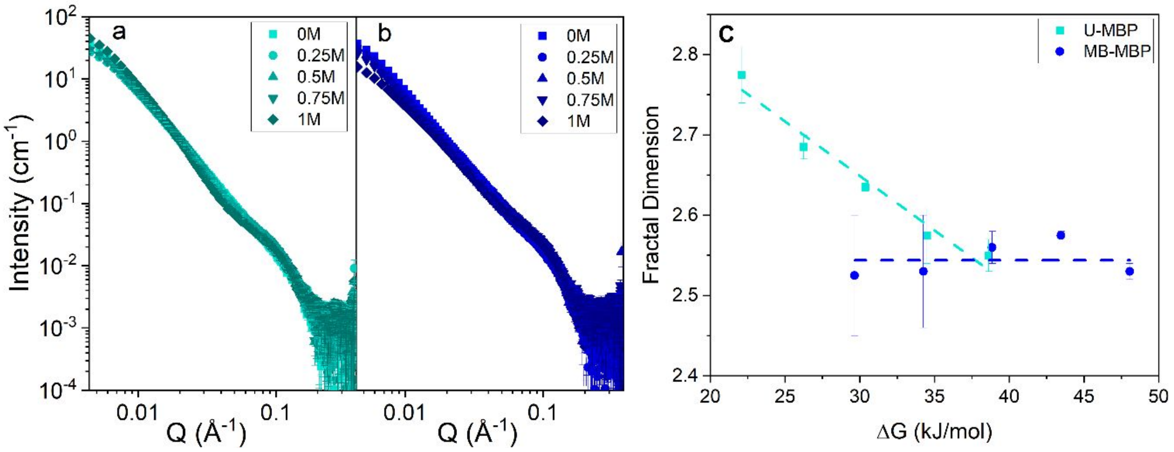

Figure S7: SAXS curves of **a)** U-MBP and **b)** MB-MBP hydrogels in varying concentrations of urea. **c)** Extracted fractal dimension of crosslinked protein clusters in unbound and maltose bound MBP hydrogels as a function of protein thermodynamic stability (ΔG_{UN}). Dashed lines shown as a guide for the eye.
